# Ethiopia towards polio eradication: insights from acute flaccid paralysis surveillance data analysis 2016 to 2023

**DOI:** 10.11604/pamj.2026.53.132.49028

**Published:** 2026-03-17

**Authors:** Geremew Tsegaye Mulugeta, Mikias Alayu Alemu, Adamu Zerihun Gelaw, Gizaw Teka Nibzane, Habtamu Tilahun Guiadie, Mesfin Tefera Manyazewal, Mesfin Wossen Getaneh, Melkamu Abte Afele

**Affiliations:** 1 Center for Public Health Emergency Management, Ethiopian Public Health Institute, Addis Ababa, Ethiopia

**Keywords:** AFP surveillance, polio eradication, data analysis, Ethiopia

## Abstract

**Introduction:**

Ethiopia remains at high risk of Circulating Vaccine-Derived Poliovirus (cVDPV2) and wild poliovirus (WPV) outbreaks due to high cross-border population movement and low population immunity. This study aims to analyze Acute Flaccid Paralysis (AFP) data from 2016 to 2023 to identify challenges in surveillance and capitalize on the lessons learned.

**Methods:**

we conducted a descriptive cross-sectional analysis of AFP surveillance data from January 2016 to December 2023. AFP is defined as the sudden onset of paralysis among children under 15 years of age, and the data were extracted from AFP case-based reports. Data were analyzed using Microsoft Excel, and QGIS 3.1.1 software was used to show the cVDPV2 hot spot areas.

**Results:**

the average annual AFP rate was 2.74 per 100,000 children under 15. Most cases (61.9%) were aged 0-5 years. Stool adequacy was 91%, and 72% were notified within seven days of onset. Investigation within two days occurred in 75% of cases. Vaccination coverage among cases was 72%. Sixty-four cases (0.6%) were cVDPV2 positive; 94.5% were discarded. Notification completeness was lowest in Tigray (55%), Afar (72%), and Oromia (76%). Benishangul-Gumuz, Oromia, Afar, Gambella, and Dire Dawa had suboptimal stool adequacy for more than 3 years.

**Conclusion:**

despite national progress in AFP surveillance, gaps persist in notification, investigation, and vaccination, especially in border and conflict-affected areas. We recommend digitalizing case management at district and facility levels, enhancing cross-border surveillance, strengthening community-based approaches, and improving routine immunization to meet WHO targets.

## Introduction

Acute flaccid paralysis (AFP) is a clinical illness characterized by the sudden onset of limb feebleness, described as flaccid among children under 15 years [1,2]. Through the efforts of the global polio eradication (GPEI) initiative since 1988, the global incidence of poliovirus has dropped by 99%, as a result, wild poliovirus (WPV) types two and three have been eradicated globally. After Nigeria achieved polio-free status, Africa was declared polio-free of indigenous WPV in August 2020 [3]. By the end of 2023, only Pakistan and Afghanistan remained endemic for WPV type 1, while Mozambique and Malawi reported imported cases. In addition, outbreaks of circulating vaccine-derived poliovirus (c-VDPV) were reported in 42 countries [3-6].

AFP surveillance through case detection, rapid investigation, sample collection, and standardized testing remains central to eradication efforts. Key performance indicators include a non-polio AFP rate of ≥2 per 100,000 children under 15 and stool adequacy ≥80%. However, GPEI faces persistent challenges due to epidemiological shifts and programmatic constraints. The COVID-19 pandemic further disrupted surveillance, causing a 34% global decline in AFP testing in 2020, rising to 46% in Africa [4-7]. By 2022, 14.3 million children globally were zero-dose. In Ethiopia, Polio 3 coverage among children aged 12-35 months was 64% in 2022 and 69% in 2023, leaving over one-third of the cohort vulnerable [8,9]. To address the gap, GPEI's 2023-2026 strategy emphasizes community engagement, government accountability, and strengthened partnerships to interrupt WPV and cVDPV transmission [5]. Surveillance is supported by environmental and iVDPV monitoring, aligned with the Global Polio Surveillance Action Plan (2022-2024) [5].

In districts characterized by high-risk, the program aims for an annual rate of over three non-polio AFP cases per 100,000 children under 15 years, alongside a national NAFP notification rate of over two cases per 100,000 population [10]. Out of the 34 priority countries identified globally, 24 are in the WHO African region, but only 19 (79.2%) met the expected NAFP and stool adequacy indicators in 2023. At the sub-national level, only 72% of these countries achieved the expected 80% target [6]. WHO recommends nationwide case-based surveillance identifying outbreaks, monitoring changes in circulating strains, progress towards eradication, determining disease burden, generating evidence on vaccine impact, and guiding optimal vaccination use [11]. All nations must report WPV or cVDPV outbreaks under International Health Regulations (IHR, 2005) and declare them as national public health emergencies [12]. AFP surveillance is critical for documenting the absence of poliovirus circulation, which is necessary for polio-free certification and maintaining the achievements [12].

Ethiopia joined GPEI in 1996 and established AFP surveillance in 1997. Coordination occurs at national, regional, and district levels through the Public Health Emergency Management (PHEM) system. PHEM focal persons lead case investigations, while health extension workers and the Health Development Army support community-based reporting [11]. However, in Africa, including Ethiopia, polio surveillance was ineffective due to a fragmented system, challenges in leveraging surveillance structures, and a vertical, disease-specific approach. Additionally, the reporting tools, timeliness of detection, notification, investigation, and overall surveillance completeness did not meet the required standards [13].

Despite four polio-free years (2017-2020), Ethiopia remains vulnerable due to cross-border movement, low vaccination in conflict zones, weak surveillance, and limited operational research. In 2016, only 66% of districts met weekly reporting completeness targets, highlighting the need for robust, sensitive surveillance [11,14]. This study analyzes Ethiopia's AFP surveillance data from 2016-2023 to identify gaps and successes, offering insights to strengthen eradication efforts and reinforce high-quality surveillance and immunization coverage.

## Methods

**Study area:** Ethiopia is situated in the northeastern region of Africa, specifically in the Horn of Africa. It borders Eritrea to the north, Djibouti and Somalia to the east, Kenya to the south, Sudan to the west, and South Sudan to the southwest. Ethiopia is the second most populous country in Africa, with a total population of around 109.8 million in 2023 and a mean age of 18.8 years. The proportion of children under five years old is estimated to be 14.6%. Administratively, the country has 12 regions and two city administrations. Ethiopia's health care system is structured around the primary health care (PHC) unit approach, which is organized into three tiers. According to the Ministry of Health (MOH) in 2024, Ethiopia's public health facilities comprised a total of 15,357 health posts, 3,907 health centers, 270 primary hospitals, 106 general hospitals, and 28 specialized comprehensive hospitals [15].

**Study design, period and population:** a descriptive cross-sectional study was employed to analyze AFP surveillance data from January 2016 to December 2023. The data abstraction was conducted from January 1 to 30, 2024. The study population included all children under the age of fifteen who manifested with AFP of any cause throughout the study period.

**Data collection procedures:** a semi-structured data abstraction checklist was developed to extract study variables from the national surveillance line list archived by the EPHI-VPD Surveillance and Response Unit, which was collected using a case-based format. Using this checklist, we extracted AFP surveillance data spanning eight years (2016-2023) from the national vaccine-preventable disease surveillance and response unit. The checklist contains variables such as sociodemographic details of the cases (age, sex, and residence area), the number of reported cases of AFP, clinical information (fever), vaccination status of the cases, stool adequacy, date of disease onset, investigation and notification dates, and final laboratory classification results. Trained personnel conducted data extraction to ensure accuracy and consistency.

**Data analysis procedure:** data were initially entered and cleaned using Microsoft Excel 2016 to ensure accuracy and consistency. The cleaned data were then exported to Power BI for comprehensive analysis, allowing for the creation of detailed visualizations and dashboards. Geographic distribution of cVDP2 cases was mapped using QGIS Version 3.3.1 software. The findings of this study were summarized using percentages, graphs, and charts.

### Standard case definition

***An AFP case:*** defined as a child under 15 years of age presenting with recent or sudden onset of floppy paralysis or muscle weakness due to any cause, or any person of any age with paralytic illness if poliomyelitis is suspected by a clinician.

***Confirmed polio case:*** a suspected case with wild poliovirus (WPV) or vaccine-derived poliovirus (VDPV) isolation from a stool sample.

***Non-polio AFP cases:*** discarded cases or all AFP cases excluding WPV and compatible cases.

***Non-polio AFP rate:*** number of non-polio AFP cases < 15 years old X 100,000/total number of children < 15 years old.

***Stool adequacy rate:*** total number of AFP cases with 2 stool specimens within 14 days of onset of paralysis * 100/total AFP cases reported.

***Discarded cases:*** non-polio AFP cases classified by the National Expert Polio Eradication Committee (NEPEC) after an in-depth review, excluding all WPV, VDPV, and compatible cases.

***Inadequate cases:*** cases detected after 14 days from the date of onset of paralysis and where the stool specimens arrived at the laboratory in poor condition.

**Data quality assurance:** to ensure data quality, the database was thoroughly reviewed for the necessary variables before the commencement of the data collection period. During the data collection process, entries were meticulously checked for completeness, and any missing values were identified and addressed. Additionally, regular consistency checks were performed to detect and correct any discrepancies. Data cleaning procedures were implemented to handle missing or inconsistent data, ensuring the dataset was accurate and reliable before data analysis.

## Results

**Demographic characteristics of the cases:** during the study period from 2016 to 2023, a total of 10,493 AFP cases were identified by the surveillance system and reported from all regions. The average annual number of reported cases was 1,311. Among the reported AFP cases, 6159 (58.7%) were males. The majority of cases, 6501 (61.9), were children aged 0-5 years, with a mean age of 5 (Std 3.78). Fever onset was reported in 7925 (75.5%) of the total cases (Table 1).

**Table 1:** demographic characteristics of the notified AFP cases from 2016 to 2023, Ethiopia

Characteristics of cases		Year								Average
		2016	2017	2018	2019	2020	2021	2022	2023	
		N (%)	N (%)	N (%)	N (%)	N (%)	N (%)	N (%)	N (%)	N (%)
Sex	Male	618(59)	622(56)	630(58)	714(58)	789(60)	1020(60)	930(60)	836(58)	6159(58.7)
	Female	430(41)	478(43)	456(42)	512(42)	551(41)	673(40)	621(40)	612(42)	4333(41.3)
Age in Years	0-5years	603(57)	649(59)	657(60)	744(61)	884(66)	1052(62)	974(63)	938(65)	6501(61.9)
	6-10years	280(27)	266(24)	262(24)	304(25)	308(23)	384(23)	373(24)	366(25)	2543(24.2)
	11-15+years	165(16)	185(17)	166(15)	178(15)	148(11)	257(15)	204(13)	144(10)	1447(13.8)
Total AFP cases reported		1048	1100	1086	1226	1340	1693	1551	1448	10,492 (1311/yr)

**Case notification, investigation and stool adequacy:** out of the 10,492 reported AFP cases, 6,571 (72%) were notified to the district health offices within seven days of the onset of AFP symptoms. The highest percentage of prompt notifications, 873 (83%), was reported in 2017, while the lowest, 1,018 (60%), was in 2021. Two stool samples were collected within two weeks of paralysis onset for 8,266 (91%) of the cases, with 8587 (95%) of the stool samples reaching the lab within 72 hours of collection and 8,450 (94%) arriving in good condition (Table 2).

**Table 2:** case notification, investigation, and stool sample management report from 2016 to 2023, Ethiopia

Indicators	Reporting year								Average
	2016	2017	2018	2019	2020	2021	2022	2023	
	N (%)	N (%)	N (%)	N (%)	N (%)	N (%)	N (%)	N (%)	N (%)
% of Cases Notified within 7 days of paralysis onset	873(83)	873(79)	814(75)	882(72)	910(68)	1018(60)	1201(77)	966(66.7)	7537 (72.0)
% of cases investigated within 2 days of notification	880(84)	937(85)	951(88)	1039(85)	1128(84)	1498(88)	1438(93)	1275(88)	7871 (75.0)
% of stool samples collected within 14 days of onset and with a 48-hour interval.	968(92)	1025(93)	1013(93)	1111(91)	1201(90)	1561(92)	1387(89)	1379(92)	9645(92.0)
% of stool samples to reach the lab within 72 hours of collection	1013(97)	997(91)	1046(96)	1172(96)	1193(89)	1645(97)	1521(98)	1391(96)	9978 (95.0)
% of stool samples to reach the lab in good condition	852(81)	1019(93)	972(90)	1164(95)	1312(98)	1640(97)	1541(99)	1425(98)	9925 (94.6)
% of AFP cases followed up at 60 days for residual paralysis identification	103(10)	85(8)	183(17)	165(13)	169(13)	157(9)	105(9)	93(6.4)	967 (9.0)
cVDPV2	0	0	0	15(1.2)	37(3)	0	1(0.06)	0	64(0.6)
Compatible cases	3(0.3)	3(0.3)	2(0.2%)	3(0.2)	3(0.2)	2(0.1)	1(0.06)	0	17(0.16)
NPENT	125(12)	99(9)	84(8)	68(6)	78(6%)	167(10)	131(8)	??9(0.6)	761(7.2)
Discarded cases	1045(99.7)	1092(99)	1033(95)	1183(96)	1293(97)	1556(91.5)	1415(91)	1305(90)	9922(94.5)
Non -AFP cases	0	5(0.45)	4(0.4%)	2(.2)	0	0	0	13	24(0.2)
Pending results	0	5(0.45)	51(5)	25(2)	7(0.5)	126(7)	134(9)	0	348(3.3)
**Total**	1048	1100	1086	1226	1340	1693	1551	1448	10492

**Final classification of cases:** of the total AFP cases reported from 2016 to 2023, 64 (0.6%) were cVDPV2 positive, 17 (0.2%) were clinically compatible cases, and 8,617 (95%) were discarded cases. The highest number of cVDPV2 cases was reported in 2020, with 37 cases (3.0%), and in 2019, with 15 cases (1.2%), from the reported AFP cases during the same period. Pending laboratory test results increased from 7 in 2020 to 134 in 2022 (Table 2).

**Figure 1:**
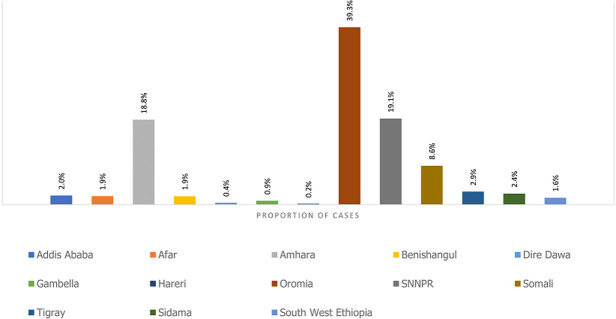
proportion of notified AFP cases by region from 2016 to 2023, Ethiopia

**Proportion of AFP cases:** the highest proportion of cases was reported from Oromia (39%) and SNNPR regions (19.1%), while the lowest proportion was reported from Dire Dawa city (0.4%) and Harari Region (0.4%) (Figure 1).

**Stool adequacy and AFP rate:** only Addis Ababa City Administration, Amhara, the late SNNPR, and Somali regions consistently achieved and maintained the AFP surveillance indicators. Benishangu-Gumuz, Afar, Gambela, Harari regions and Dire Dawa City administration did not achieve stool adequacy in at least one reporting year. Additionally, Harari in 2018, Gambela in 2020, and Afar in 2022 and 2023 did not achieve the NP-AFP rate, respectively. The Tigray region did not maintain the NP-AFP rate for three consecutive years (2020-2022) as described (Table 3).

**Table 3:** acute flaccid paralysis case detection and stool adequacy rate, 2016-2023, Ethiopia

S.N	Year	2016		2017		2018		2019		2020		2021		2022		2023		Average	
	Region name	AFP rate	Stool adequacy	AFP rate	Stool adequacy	AFP rate	Stool adequacy	AFP rate	Stool adequacy	AFP rate	Stool adequacy	AFP rate	Stool adequacy	AFP rate	Stool adequacy	AFP rate	Stool adequacy	AFP rate	Stool adequacy
1	Addis Ababa	2.1	94	2.4	90	2.6	91	3.1	100	3.6	84	3.5	93	3.1	97	3.4	94	2.98	92.9
2	Afar	3.1	88	2.4	95	3.5	96	3.0	81.0	3.0	100	2.4	96	1.6	71	1.9	90	2.80	89.6
3	Amhara	2.7	94	2.7	95	2.5	95	2.3	91	2.4	93	3.0	94	3.2	94	2.0	96	2.78	94.0
4	B/ Gumuz	2.4	83	4	89	5.8	86	4.3	81	2.7	75	4.0	89	5.3	86	4.4	100	3.99	86.1
5	Dire Dawa	2.7	50	2	100	2.7	100	2	100	3.5	88	5.5	100	3.0	83	2.5	80	3.10	87.6
6	Gambela	2.5	100	5.5	91	7.5	87	6.9	83	1.7	100	3.7	85	2.6	100	2.0	75	4.23	90.1
7	Hareri	2	50	3	100	1.0	100	3.0	100	2.0	100	5.0	80	4.0	100	2.0	100	2.93	91.3
8	Oromia	2.3	89	2.4	88	2	88	2.3	88	2.9	86	3.4	90	2.8	91	2.8	95	2.69	89.4
9	SNNPR	2.5	94	2.6	95	2.5	96	2.6	95	2.8	93	4.1	94	3.6	95	2.4	94	3.01	94.5
10	Somali	3.7	92	3.3	94	3.2	92	4.7	92	4.5	93	4.1	95	3.1	98	3.4	96	3.75	94.0
11	Tigray	2.4	82	2.1	93	2	80	2.2	92	1.3	97	0.4	100	0.2	100	2.4	98	1.75	92.8
12	Sidama	-	-	-	-	-	-	-	-	-	-	3.6	90	3	94	2.6	95	3.33	93.0
13	SWE	-	-	-	-	-	-	-	-	-	-	-	-	4.6	95	4.6	96	4.00	95.5
	National	2.6	83.3	2.9	93.6	3.2	91.9	3.3	91.2	2.8	91.7	3.6	85.1	3.1	92.6	2.8	93	3.11	90.3

**Figure 2:**
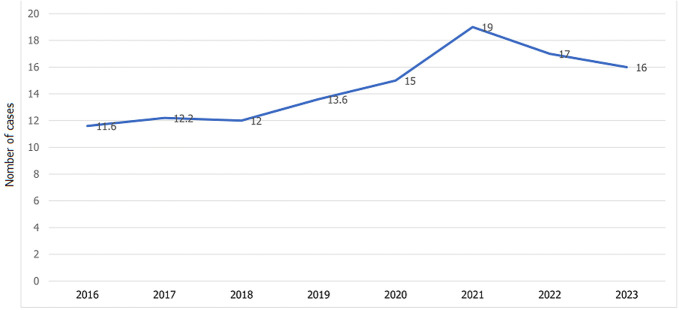
trend of reported NP-AFP cases from 2016 to 2023, Ethiopia

**NP-AFP trend:** the highest proportion of AFP cases was reported in 2021 and 2023, with 1,693 (19%) and 1,551 (17%) respectively. In contrast, the lowest number of cases was reported in 2016, with 1048 cases (11.6%). The trend of NP - AFP rate from 2016 to 2023 showed a decreasing trend in 2023 by 142 cases (8.4%) compared to 2021 (Figure 2).

**Figure 3:**
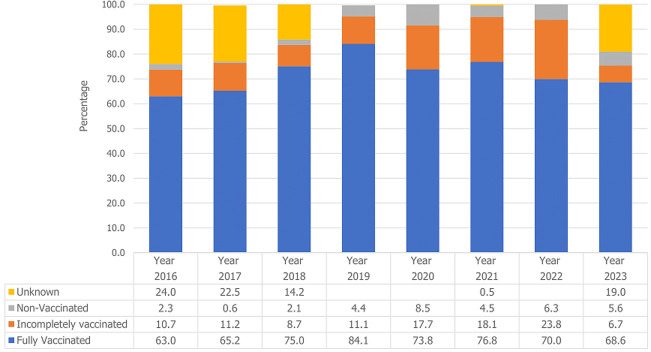
vaccination status of the notified AFP cases from 2016 to 23, Ethiopia

**Vaccination status:** regarding the vaccination status of the cases, around 500 (4.9%) of the reported NP- AFP cases were unvaccinated and 1475 (14%) of them were incompletely vaccinated against the poliovirus. Throughout the study period, the number of non-vaccinated cases varied, with the highest at 114 (9%) in 2020 and the lowest at 25 (2.3%) in 2017. Incompletely vaccinated cases (less than 2 doses) showed a significant increase, with the highest proportion, 369 (24%) of incompletely vaccinated cases, reported in 2022 (Figure 3).

**Figure 4:**
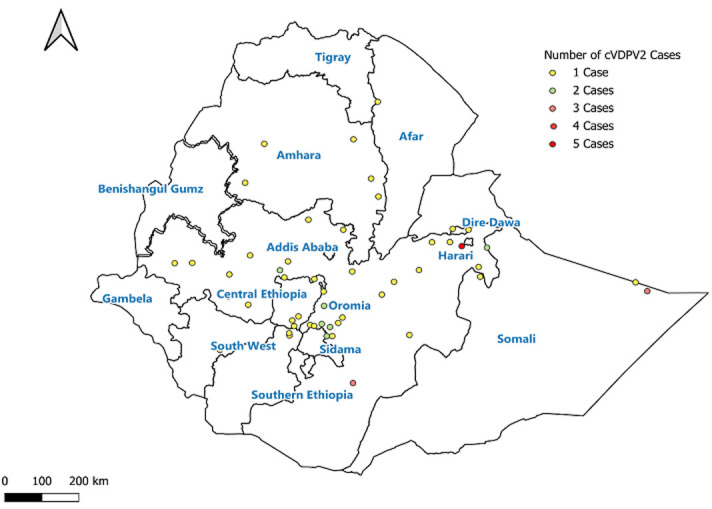
distribution of cVDPV2 cases by district from 2016 to 2023, Ethiopia

**Geographical distribution of vaccine-derived poliovirus 2 in Ethiopia:** seven regions reported cVDPV2 confirmed cases, with Oromia regional state having the highest proportion at 40 cases, accounting for 63% of the total. Additionally, 17 clinically compatible cases were reported from seven regions, with Oromia and Somali regions having the highest proportion of these cases, each contributing 29% to the total (Figure 4).

**Reporting completeness:** the regions with the highest completeness by districts are Somali, Gambela, and Southwest Ethiopia (SWE), each with an average of 114% to 115%. On the other hand, regions like Tigray, Afar, and Oromia had the lowest average completeness rates, with Tigray at 55%, Afar at 72%, and Oromia at 76%, suggesting potential gaps in AFP surveillance (Table 4).

**Table 4:** average of districts that notified at least one AFP case per year per region, 2016 to 2023, Ethiopia

Year	Regional	Addis Ababa	Afar	Amhara	B/Gumz	DIire Dawa	Gambela	Harari	Oromia	SNNPR	Somali	Tiigray	Sidama	SWE
		N (%)	N (%)	N (%)	N (%)	N (%)	N (%)	N (%)	N (%)	N (%)	N (%)	N (%)	N (%)	N (%)
2016	Expected	10	16	131	9	1	3	1	302	135	43	38	-	-
	Reported	8(80)	15(94)	139(106)	9(100)	1(100)	2(75)	1(100)	226(75)	108(12)	47(10)	32(8)	-	-
2017	Expected	10	17	129	14	1	4	1	241	135	46	37	-	-
	Reported	7(70)	10(59)	107(93)	10(71)	1(100)	7(180)	1(100)	169(70)	122(97)	44(95.5)	25(67)	-	-
2018	Expected	10	16	132	12	1	4	1	241	139	51	38	-	-
	Reported	8(80)	15(94)	102(77)	13(92)	1(100)	8(200)	1(100)	170(70)	123(88)	53(102)	27(71)	-	-
2019	Expected	10	21	118	20	1	7	1	249	136	54	36	-	-
	Reported	10(100)	18(86)	86(73)	16(80)	1(100)	10(142)	1(100)	170(68)	114(82)	70(129)	28(78)	-	-
2020	Expected	10	21	118	20	1	7	1	249	136	54	38	-	-
	Reported	10(100)	15(71)	89(75)	16(80)	1(100)	5(71)	1(100)	202(81)	111(96)	72(130)	22(58)	-	-
2021	Expected	10	21	148	14	1	7	1	299	131	60	41	31	-
	Reported	10(100)	15(71)	119(80)	12(86)	1(100)	6(90)	1(100)	256	126(96)	66(110)	11(27)	27(87)	-
2022	Expected	11	21	147	11	1	7	1	302	105	62	41	31	27
	Reported	11(100)	8(38)	130(88)	12(109	1(100)	7(100)	1(100)	238(79)	100(95)	68(109)	2(5)	30(97)	31(115)
2023	Expected	11	21	148	11	1	8	1	303	105	63	41	32	27
	Reported	11(100)	15(71)		11(100	1(100)	5(63)	1(100)	232(77)	83(79)	67(106)	35(85)	28(88)	30(111)
Total districts expected to notify at least 1 AFP case		71	133	923	100	7	39	7	1883	917	370	269	62	27
Total districts that reported at least 1 AFP case and %		64(90)	96(72)	772(84)	88(88)	7	45(115)	7(100)	1431(76)	804(88)	420(114)	147 (55)	57(92)	31(115)

## Discussion

From 2016 to 2023, the surveillance system in Ethiopia identified 10,492 NP-AFP cases, with a case detection rate of 3.11/100,000 among children under 15 years and a stool adequacy rate of 91%. Of these, 8,617 (95%) were discarded, 64 (0.6%) were cVDPV2 positive, and 17 (0.2%) were clinically compatible cases. The highest proportion of AFP cases was reported from Oromia (39%), SNNP (20%), and Amhara (19%) regions. Only 72% of cases were notified to district surveillance officers within seven days of paralysis onset, and 78% were investigated within two days of notification. Over half (61.5%) of the reported cases were children under five years old, consistent with previous studies conducted in Ethiopia, Zambia and Niger [16]. AFP and Guillain-Barre Syndrome (GBS) share similar clinical features and disproportionately affect young children [17]. This finding is further supported by another study conducted in Ethiopia, which showed that 68.8% of GBS patients were under five [18]. These results underscore the importance of early diagnosis and targeted surveillance of APP-related symptoms and diseases in children under five years.

Ethiopia has made significant strides towards polio eradication over the past eight years, achieving an average stool adequacy rate of 90.3% and an NP-AFP notification rate of 3.11% nationally. Compared to previous local studies [16], these findings indicate a notable improvement and are comparable with studies conducted in Niger [19]. This progress has been achieved through coordinated program management and financial support from both government and non-governmental agencies. Despite the success registered in polio eradication efforts, cVDPVs have emerged in areas with low population immunity, posing a risk of international spread and paralysis [20]. Ethiopia detected the first cVDPV2 positive cases in 2019, with a total of 64 cases reported by 2023. The majority of these cases (56%) were reported in 2020, followed by 15% in 2019. Globally, from 2018 to 2023, a total of 1,352 children were paralyzed due to c VDPV, with the highest number of cases (1,113 or 82.3%) reported in 2020 [21]. In 2023, neighboring countries of Ethiopia, including Kenya (8 cases), South Sudan (8 cases), and Somalia (3 cases), confirmed cVDP2 cases, indicating a high risk of polio importation due to cross-border population movements [16,19]. Effective collaboration between neighboring countries is essential to strengthen surveillance and response systems, preventing polio importation into Ethiopia.

Ethiopia met the annual target of reporting two non-polio acute flaccid paralysis (AFP) cases per 100,000 children under 15 years, marking a significant milestone in polio eradication. This finding is consistent with studies conducted in Ethiopia and other African countries [16]. However, to further enhance surveillance and response, it is recommended to increase the annual rate of non-polio AFP cases to at least three cases per 100,000 children under 15 years in regions identified as high-risk or experiencing outbreaks like Ethiopia [10].

During the study period, the average completeness of districts reporting at least one AFP case per 100,000 population per year was not achieved in the Tigray, Amhara, and Oromia regions, indicating gaps in AFP surveillance completeness at the sub-regional level. In 2023, eight of 28 (29%) countries at high risk for poliovirus spread failed to meet global AFP surveillance indicator targets at the sub-national level [22]. Challenges such as competing outbreaks, inaccessibility to insecure districts, and non-integrated health delivery systems hinder the implementation of planned polio eradication activities [12]. Polio surveillance does not meet the minimum recommended eradication standards in many of the WHO regions at sub national level, limiting evidence-based decision-making for the eradication program [23]. Silent districts, characterized by low rates of case detection, are susceptible to the circulation of undetected cases, thereby hindering timely response efforts.

Achieving a consistent stool adequacy rate of greater than 90% and an NP-AFP notification rate at the sub-national level remains a significant challenge, particularly in high-risk regions such as Tigray, Afar, and Benishangul Gumuz. These regions are susceptible to polio importation from neighboring countries, which exacerbates the difficulty in maintaining robust surveillance and vaccination coverage. Similar challenges have been observed in other high-risk countries. For instance, a study conducted in Zambia and Niger from 2015 to 2021 highlighted the struggle to maintain high stool adequacy rates, which ranged from 70.9% to 90.2% [20].

The experience of other countries also highlights the impact of sub-national gaps in surveillance. A report on polio eradication efforts worldwide during 2020-2021 found that while national AFP surveillance performance improved in many priority countries, substantial gaps persisted at the sub-national level [24]. These gaps can lead to delayed detection and response to poliovirus transmission, increasing the risk of outbreaks.

Achieving consistent high-quality routine vaccination coverage of greater than 95% is crucial for polio eradication. However, only three-fourths of reported AFP cases were fully vaccinated, comparable to previous studies in Ethiopia [16]. Globally, routine vaccination coverage has declined, particularly in low and middle-income countries. WHO records show routine vaccination coverage dropped from 86% in 2019 to 81% in 2021 [25]. This decline in routine immunization coverage contributed for pooled susceptibility and increase risk of poliovirus outbreaks and importation of cases [12].

In Ethiopia, the situation is further complicated by the fact that approximately 26% of notified AFP cases were not fully vaccinated against the poliovirus. This gap in vaccination coverage hinders the achievement of herd immunity, leaving the population vulnerable to outbreaks. The importance of high vaccination coverage is underscored by global standards, which recommend a routine vaccination coverage of at least 95% to ensure herd immunity and prevent the spread of poliovirus [26]. The gaps in routine immunization (RI) implementation are attributed to several factors, including a fragile immunization program, compromised quality of polio supplementary immunization activities (SIA), civil conflict and insecurity, and issues with health service accessibility, particularly among high-risk groups such as pastoralists [21].

Despite the Oral Polio Vaccine (OPV)'s success in eliminating 99% of polio cases since 1988, a new strain of the poliovirus that has genetically changed from its original form in the oral polio vaccine (OPV) is being reported. This mutated virus can spread and cause paralytic polio, especially in areas with low immunization coverage [27]. This phenomenon has been a significant challenge in the global effort to eradicate polio. In the early 2000s, many countries transitioned from using OPV to the Inactivated Polio Vaccine (IPV), which does not carry the risk of cVDPV. Despite this switch, sporadic cases of cVDPV continue to be reported in regions with community-level immunity gaps showing a gap in routine immunization [28].

Ethiopia shares extensive geographical boarders with neighboring countries and has approximately 127 designated points of entry [29]. The population residing in border areas frequently crosses these borders for various purposes, sometimes with their animals [30]. Effective cross-border collaboration and coordinated One Health approaches are essential to prevent polio importation and maintain the achieved polio eradication goals. This requires strengthening surveillance and response systems at local points of entry [30].

The World Health Organization's (WHO) International Travel and Health guidelines recommend that all travelers to polio-affected areas be fully vaccinated against polio. Residents and visitors staying for more than 4 weeks in infected areas should receive an additional dose of the oral polio vaccine (OPV) or inactivated polio vaccine (IPV) within 4 weeks to 12 months of travel. However, there is no effective mechanism for controlling land border crossings in Ethiopia, posing a significant risk of polio importation [31].

The strengths of this study lie in the inclusion of a large AFP sample size, which highlights the epidemiological distribution of AFP, its surveillance performance indicators, and gaps in Ethiopia from 2016 to 2023. Other strengths of the study include the triangulation of age, geographical area, vaccination status, and stool condition with AFP case findings. This study may exclude some clinical variables, which affect the comprehensiveness of the findings and the ability to fully understand the clinical aspects of the reported AFP due to the use of secondary data.

**Recommendations:** to strengthen polio eradication efforts in Ethiopia, it's crucial to intensify AFP surveillance, especially in high-risk and hard-to-reach regions. Surveillance at points of entry should be expanded to monitor and control the movement of potentially infected individuals, while cross-border collaboration with neighboring countries is vital to synchronize vaccination efforts and share data effectively. Digitalizing case notification and investigations at district and health facility levels will allow for real-time AFP data management, improving responsiveness and data quality.

Applying a One Health approach, alongside improving health service access in insecure and remote areas, helps ensure integrated disease management. Community-based surveillance should be remodeled to enable early detection of suspected cases, complemented by robust monitoring and evaluation of polio program performance. Immunization activities must be strengthened through improved routine coverage, catch-up campaigns, and requirements for travelers from high-risk countries to be vaccinated at points of entry. A revised outbreak response strategy for cVDPV2s should be implemented, and humanitarian actors must prioritize child vaccination in conflict zones and border regions as part of essential health services.

## Conclusion

This study highlights the significant progress Ethiopia has made towards polio eradication, evidenced by the high stool adequacy rates and NP - AFP notification rates achieved nationally. Despite these achievements, there are notable gaps in report completeness and at the sub-regional level, particularly in conflict-affected and border regions. The number of cases notified within seven days of paralysis onset and investigated within two days of the notification was not satisfactory compared to the polio eradication standard. The high proportion of AFP cases in children under five years old emphasizes the importance of targeted surveillance and early diagnosis in this vulnerable age group. Vaccination coverage among cases lags behind the World Health Organization's 95% goal, resulting in a significant number of children being infected with cVDPV2 in many regions of the country. The persistence of circulating cVDPV2 cases and the challenges in maintaining high routine immunization coverage underscore the need for continued vigilance and enhanced surveillance efforts.

### What is known about this topic

- A sensitive acute flaccid paralysis (AFP) surveillance remains a cornerstone of the global polio eradication strategies;
- Circulating vaccine-derived poliovirus type 2 (cVDPV2) continues to cause paralysis, among partially or unvaccinated children in low-resource settings;
- Achieving and sustaining ≥95% coverage with three doses of polio vaccine is essential to protect children and interrupt virus transmission.

### What this study adds

- Of the reported cases, 72% were notified within seven days of paralysis onset, and 75% were investigated within two days of notification, reflecting timely response in the surveillance system;
- During the study period, 64 cases of circulating vaccine-derived poliovirus type 2 (cVDPV2) were reported; over one-fourth of the reported cases had incomplete polio vaccination histories;
- Immunization alone is not sufficient to control imported cases or minimize the risk of importation. Implementing an effective mechanism to screen the polio vaccination status of individuals crossing land borders is essential.
